# Cardioprotective effect of silicon-built restraint device (ASD), for left ventricular remodeling in rat heart failure model

**DOI:** 10.1007/s10856-022-06663-6

**Published:** 2022-05-10

**Authors:** Waqas Nawaz, Muhammad Naveed, Jing Zhang, Sobia Noreen, Muhammad Saeed, Kiganda Raymond Sembatya, Awais Ullah Ihsan, Imran Shair Mohammad, Gang Wang, Xiaohui Zhou

**Affiliations:** 1 Department of Clinical Pharmacy, School of Basic Medicine and Clinical Pharmacy, China Pharmaceutical University, Nanjing, China; 2 Department of Clinical Pharmacology, School of Pharmacy, Nanjing University, Nanjing, China; 3 Department of Pharmaceutics, Faculty of Pharmacy, The Islamia University of Bahawalpur, Bahawalpur, Pakistan; 4 The Cholistan University of Veterinary and Animal Sciences, Bahawalpur, Pakistan; 5 School of Pharmaceutical Sciences, Sun Yat-Sen University, Guangzhou, China; 6 Department of Heart Surgery, Nanjing Shuiximen Hospital, Nanjing, China; 7 Department of Cardiothoracic Surgery, Zhongda Hospital affiliated with Southeast University, Nanjing, China

## Abstract

This study aims to evaluate the feasibility and cardio-protective effects of biocompatible silicon-built restraint device (ASD) in the rat’s heart failure (HF) model. The performance and compliance characteristics of the ASD device were assessed in vitro by adopting a pneumatic drive and ball burst test. Sprague-Dawley (SD) rats were divided into four groups (*n* = 6); control, HF, HF + CSD, and HF + ASD groups, respectively. Heart failure was developed by left anterior descending (LAD) coronary artery ligation in all groups except the control group. The ASD and CSD devices were implanted in the heart of HF + ASD and HF + CSD groups, respectively. The ASD’s functional and expansion ability was found to be safe and suitable for attenuating ventricular remodeling. ASD-treated rats showed normal heart rhythm, demonstrated by smooth -ST and asymmetrical T-wave. At the same time, hemodynamic parameters of the HF + ASD group improved systolic and diastolic functions, reducing ventricular wall stress, which indicated reverse remodeling. The BNP values were reduced in the HF + ASD group, which confirmed ASD feasibility and reversed remodeling at a molecular level. Furthermore, the HF + ASD group with no fibrosis suggests that ASD has significant curative effects on the heart muscles. In conclusion, ASD was found to be a promising restraint therapy than the previously standard restraint therapies.

**Figure:**
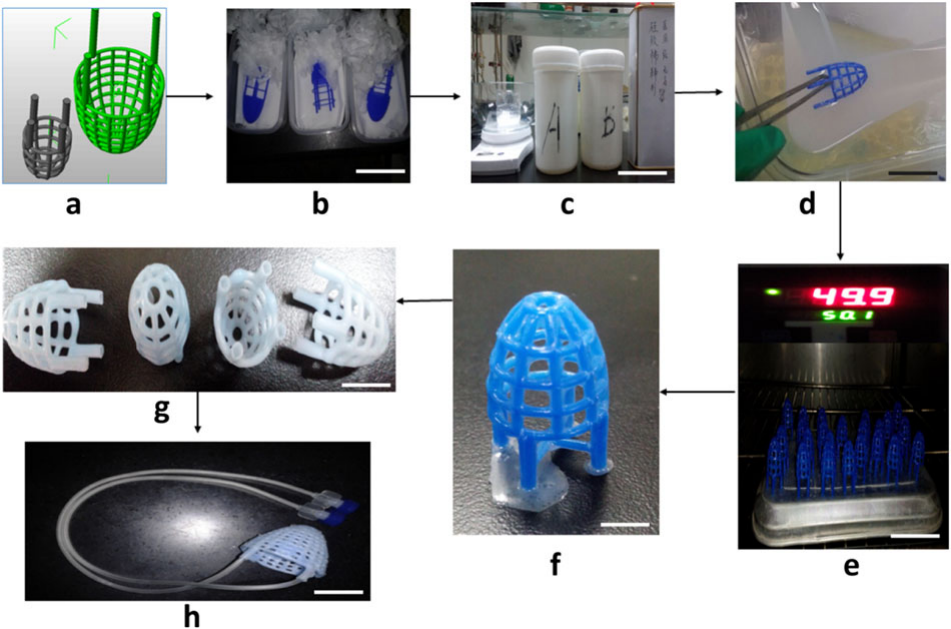
Stepwise ASD fabrication process (a) 3D computer model of ASD was generated by using Rhinoceros 5.0 software (b) 3D blue wax model of ASD (c) Silicon was prepared by mixing the solutions (as per manufacturer instruction) (d) Blue wax model of ASD was immersed into liquid Silicon (e) ASD model was put into the oven for 3 hours at 50 °C. (f) Blue wax started melting from the ASD model (g) ASD model was built from pure silicon (h) Two access lines were linked to the ASD device, which was connected with an implantable catheter (Port-a-cath), scale bar 100 µm. (Nikon Ldx 2.0).

Stepwise ASD fabrication process (a) 3D computer model of ASD was generated by using Rhinoceros 5.0 software (b) 3D blue wax model of ASD (c) Silicon was prepared by mixing the solutions (as per manufacturer instruction) (d) Blue wax model of ASD was immersed into liquid Silicon (e) ASD model was put into the oven for 3 hours at 50 °C. (f) Blue wax started melting from the ASD model (g) ASD model was built from pure silicon (h) Two access lines were linked to the ASD device, which was connected with an implantable catheter (Port-a-cath), scale bar 100 µm. (Nikon Ldx 2.0).

## Introduction

Heart failure (HF) is a devastating disorder that leads to an inadequate supply of blood to tissues and organs to meet its metabolic demands. Numerous factors can contribute to HF pathogenesis, such as myocardial infarction (MI), valvular heart disease (VHD), hypertension, and cardiomyopathy; its hallmarks include hypertrophy, loss of myocytes, and increased interstitial fibrosis [1]. HF is a global health problem that becomes worse as the population ages [2, 3]. During the last three decades, HF treatment by devices significantly improved the survival rate [4, 5] and mean life expectancy among HF patients [6]. However, despite the advancement in therapies, the mortality rate (five years) due to HF is nearly 50% more than any type of cancer [7]. Ventricle restraint therapy (VRT) is a well-established and promising approach for managing advanced-stage dilated HF. VRT consists of devices made of biocompatible materials that play a supportive role for heart muscles without having direct contact with the blood [8, 9]. The functioning of these devices was extensively investigated and studied even at different clinical phases [9–11]. However, although these devices have a long history of investigation, they are still not used in clinical practice [12, 13]. Recently, the researchers are focusing more on improving the nature of the restraint and the biocompatibility of VRT so that the left ventricular (LV) remolding in the dilated heart could be reversed [14, 15]. Left ventricular remodeling is when mechanical, neurohormonal, and possibly genetic factors alter ventricular size, shape, and function [1]. The earlier VRT devices restraint the dilated heart muscles up to the subjective level. However, the influence of restraint nature, mesh tubular design, and biocompatibility of VRT devices has not been thoroughly investigated. The current study modified VRT into exo-organoplasty intervention [16] by designing a novel ASD device made by silicon, a common and highly biocompatible material [17, 18]. The objective of the ASD device was to fill the tubules with characteristic fluids for developing restraint.

**Fig. 1:**
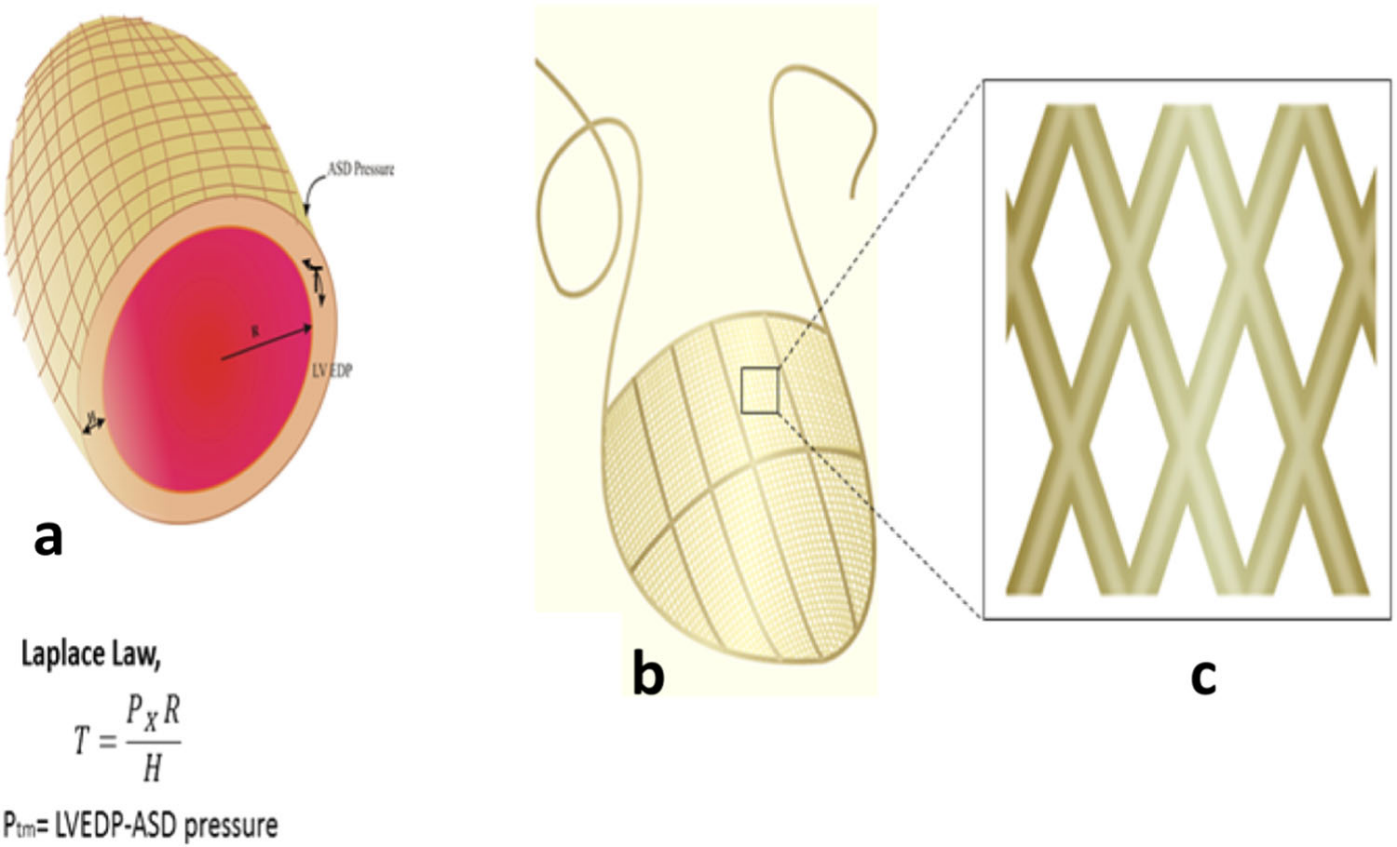
Laplace’s law explains the dilated ventricles mechanism and provides a framework for ventricle remodeling. (**T**) is the dilated heart, directly proportional to left ventricular end-diastolic pressure (LVEDP) (**P**) and radius chamber radius (**R**), inversely by wall thickness. Laplace law explains the concept of ASD’s effect on ventricle remodeling. The relationship between transmural pressure (P_tm_) and LVEDP is defined by an equation, as shown above in the figure. **a** By cross-sectional view, the ASD device covered both ventricles from base to apex. **b** ASD overview of the epicardium. The two tubules are subcutaneously extended, tunneled outside the body, and connected to a medical device for loading and releasing fluid. **c** View of ASD tubules intercommunicating with each other

Furthermore, this system might be useful to deliver pharmacological and biological agents to the heart locally [19, 20]. In this study, the following important questions have been addressed. First, assess the feasibility of a silicon-built restraint device for attenuating ventricular remodeling in rat’s model of HF? Second, whether hydraulic restraint therapy works more promisingly than former standard CSD restraint therapy or not? Active hydraulic ventricular attaching support system (ASD) comprises a net cover with hollow flexible tubules, which are intercommunicated. The ASD device covers both ventricles, and two tubules are tunneled outside to connect ASD with desired medical equipment [21]. The ASD system was designed to deliver ventricle restraint, pharmacological, and biological therapeutic agents locally to heart muscle [22–24]. The ASD tubules can be filled with fluid, which exerts continuous pressures on the dilated ventricles [25] (Fig. 1). The Laplace law provides a framework for defining means of ventricle remodeling. Ventricular wall stress can be reduced by decreasing transmural pressure (P_tm_), reducing cardiac chamber radius, promoting more exceptional ventricular wall thickness, or combining the three [26]. The function of ASD is to reduce ventricular wall stress by providing counter-pressure, as shown in Fig. 1. It can exert a continuous elastic force to the heart throughout the cardiac cycle, not just at end-diastole.

Furthermore, the current study was designed to investigate the compliance and functional characteristics of ASD in vitro. Here, the feasibility, biocompatibility, and nature of the ASD device’s restraint were studied in the rat’s HF model. It was observed that ASD is feasible and highly biocompatible. Thus, it provided an adjustable and measurable ventricular hydraulic restraint (AMVR), which could be a promising therapy to treat dilated HF.

## Materials and methods

Male Sprague-Dawley (SD) rats (240–260 g) were purchased from the College of Veterinary Medicine, Yangzhou University (License # SCXK (Su) 2015–2005), Yangzhou, China. Silk sutures and 6.0 polypropylene sutures were bought from Shanghai Jinhuan Chemical Co., Ltd., Shanghai, China. Ventilator (HX-300S), BL-420 multi-channel physiological signal system, and ECG were obtained from Chengdu technology & market Co., Ltd, Chengdu, China. The polyethylene catheter and implantable catheter were purchased from Jiangxi Hongda Medical Equipment Group Co., Ltd, Nanchang, Jiangxi, China. Biebrich scarlet-acid fuchsin kits and trichrome’s staining kits were purchased from Beyotime Institute of Biotechnology, Haimen, China. Rat BNP ELISA kits were obtained from Shanghai Jinma Biological Co., Ltd, Shanghai, China. CSD was purchased from Ethicon, Inc, Somerville, NJ USA. The animal’s ethics committee approved the protocol for this study of China Pharmaceutical University under the guidelines published by the research council by a school of pharmacy, China pharmaceutical university and it conforms to the instructions for the care and use of laboratory animals published by US national institutes of Health (NIH, 1996). All surgery was performed under pentobarbital sodium, and all efforts were made to minimize suffering.

### ASD device fabrication

The ASD comprises a silicon-based material with hollow flexible tubules intercommunicated with each other [27]. The ASD device covers both ventricles, and two tubules are tunneled outside for connecting ASD with desired medical equipment. The objective of the ASD device was to fill the tubules with characteristics fluid to deliver restraint. Furthermore, this system might be useful in providing pharmacological and biological agents to the heart locally. A three-dimensional computer model of ASD was developed by using Rhinoceros 5.0 software. The blue wax model of ASD was printed by using 3D printing technology. The blue wax model of ASD was immersed in liquid silicon and was dried in an oven at 50 °C (3 h). The wax got melted from the ASD, and the silicon-built ASD model was obtained. Leak testing (Cincinnati Test Systems, Shanghai, China) was performed to ensure any leakage and blockade in ASD tubules.

### Animal model

A total of 24 male SD rats were randomly divided into four groups (*n* = 6): control group, HF group, HF + CSD group, and HF + ASD group. HF model was established in all groups except the control group by the LAD ligation method, as previously described [28]. Before anesthesia, all the rats were shaved, weighed, disinfected, and scrubbed with 75% ethanol from the neck and chest area and anesthetized by intraperitoneal injection of 3% Phenobarbital sodium (30 mg/kg). Anesthetized rats were placed in a supine position, and endotracheal intubation was performed. Ventilation was provided by connecting the endotracheal tube to an HX-300S ventilator at a breathing rate of 80/min and tidal volume of 10 mL. The heart was exposed through a left thoracotomy via the 3rd and 4th interspaced rib resection. After an opening of the pericardium, the coronary anatomy was inspected to determine the proximal LAD coronary artery. The LAD is then transiently ligated [29] with a 6-0 polypropylene suture. The incision was closed in layers, and a chest tube was placed to drain the chest and evacuate the pneumothorax. The chest tube was removed once the animal became ambulatory. HF, defined as ST-elevation or depression, was confirmed by ECG and BNP levels. Penicillin sodium 5wU/100 g was injected intramuscularly as prophylaxis once daily for five days. In the case of treatment groups, first, the device was implanted, and the chest was closed for further experiments.

### ASD device implantation

After confirming the HF model (a method described above), ASD was implanted in the HF + ASD group rats [20]. After steady-state baseline measurement, the ASD was placed around the heart and sutured to the AV groove using prolene sutures (4–0) to cover both ventricles. The ASD device has two access lines, which are connected with implantable catheters. The ASD port-a-cath was tunneled subcutaneously through the second intercostal space into the left anterior chest wall and extended outside the body through a 1 cm opening made in the skin at spinotrapezius. The sternum and port-a-cath incision were then closed in layers. Saline was instilled in 20 mL increments into ASD tubules, and an electrocardiogram was monitored. AMVR was defined by the maximum pressure applied by the ASD to the epicardium through a constant saline volume inside the ASD tubules. Maximum ASD pressure occurred at end-diastole (the time point when the heart volume is large).

### CSD device implantation

CSD is made of polypropylene mesh and used as a positive control. CSD is a previous restraint device for delivering standard restraint and was extensively investigated in animal models and clinical trials. After establishing the HF model, the CSD device was implanted to the heart of the HF + CSD group by wrapping around the heart and secured to the AV groove. The electrocardiogram was monitored by guidelines for placement of the Acorn CorCap cardiac support device (Acorn Cardiovascular, Inc, St Paul, Minn) such that LVEDP decreased by 5% [10, 30].

### In vitro experiments

#### Functional characteristics of ASD device

This study was conducted by adopting the standard protocol of Kung et al. [31]. The functional attributes of ASD were determined using the pneumatic drive (P-Drive), and a hydraulic pump was used for the implantable system. The ASD was attached to a cylindrical, flexible tube with a diastolic diameter of 6 cm and a length of 5 cm and all system is linked to the fluid reservoir for volume compensation.

#### Compliance characteristics of ASD device

According to the manufacturer’s instructions, the compliance characteristics of ASD were determined by adopting the ball burst test (TMI Trading Shanghai Co., Ltd, China). Briefly, the pressure was applied by pressing a ball against the ASD tubules within a circular fixture. The ASD was attached around the edge of this circular fixture through a pneumatic clamping device. ASD distorted in multiaxial behavior when the pressure was applied. The transducer recorded the load at which ASD fails to withstand the pressure. Furthermore, the uniaxial compliance curve for the ASD model was drawn in the longitudinal and circumferential directions, and the multiaxial compliance curve was used for comparison.

### In vivo experiments

#### Electrocardiography

Rats were sedated with 10% chloral hydrate and placed in a supine position on a surgical table. The electrocardiogram BL-420 physiological signal acquisition system was used to analyze ECG and was recorded on a personal computer Electrocardiogram has four electrodes inserted into the rat’s limbs subcutaneously. The red electrode was inserted into the right upper limb, black into the right lower limb, yellow into the left upper limb, and green into the left leg (according to manufacturer’s instruction). Pre- and post-ligation, ECG was recorded of all rats. Post-ligation ECG was recorded on the 7th, 15th, and 30th days.

#### Hemodynamic parameters

With a steady breath, the right carotid artery was distally ligated through a longitudinal incision on the right side of the neck and fixed to prevent excessive bleeding. One end of the polyethylene catheter was inserted into the LV through the right carotid artery, and the other end was linked with a BL-420 multi-channel physiological signal system. At the end of the study period (on the 30th day), all the hemodynamic parameters were measured, like left ventricular systolic pressure (LVSP), left ventricular end-diastolic pressure (LVEDP), dp/dt_max_, and −dp/dt_max_, and mean data was recorded. Heart rate was also taken digitally through the BL-420 multi-channel physiological signal system on preoperative, postoperative, 7th, 15th, and 30th days and mean data was calculated at the end of the study period.

#### BNP measurement

B-type natriuretic peptide serum concentration was measured by the ELISA technique using rat’s BNP kit following the manufacturer’s instructions. Briefly, the rat’s blood was collected and centrifuged at 2000 rpm/min for 2 min at 4 °C, and the supernatant (blood plasma) was obtained. The plasma samples were diluted 1:4 with an example, a stop solution was added, and then the BNP was measured.

#### Histopathology

After completing the observation period (30 days), rats were euthanized, and skin and muscle tissues were excised up to the neck region from the xiphoid. The chest plate was removed along the muscles and ribs to expose the heart. The aorta was tied with a silk suture to avoid bleeding, and the heart was harvested. The heart was perfused in PBS (1:100) to flush out any remaining blood and fixed in 10% formalin. The heart was sliced in coronal sections into 5 µm, dehydrated with ascending ethanol series and embedded in paraffin. Masson’s trichrome staining of fixed samples was used to differentiate between collagen and muscle fibers. Trichrome staining was accomplished by applying Weigert iron hematoxylin followed by Biebrich scarlet-acid fuchsin (plasma stain), phosphomolybdic-phosphotungstic acid, and aniline blue (fiber stain) on 5 µm sliced section of heart on the slides [32]. Slides were finally observed under the photonic microscope with a DFC420 camera fitted for histopathological changes. Images from the sections were captured digitally by using Image Manager Software. The degree of fibrosis was quantified by using ImageJ software 4.7.0.

### Data analysis

All hemodynamic data has been presented as mean ± SEM (*n* = 3); one-way analysis of variance (ANOVA) analysis followed by Dunnett’s Multiple Comparison Test was applied for the statistical significance of other groups compared to the control group. While BNP results are expressed as mean ± SD, two-way ANOVA followed by the Bonferroni test was used for statistical analysis compared to the HF group. Where, ^*^P = 0.05, **P < 0.01, ***P < 0.001.

## Results

### In vitro experiments

#### Functional characteristics of ASD device

**Fig. 2:**
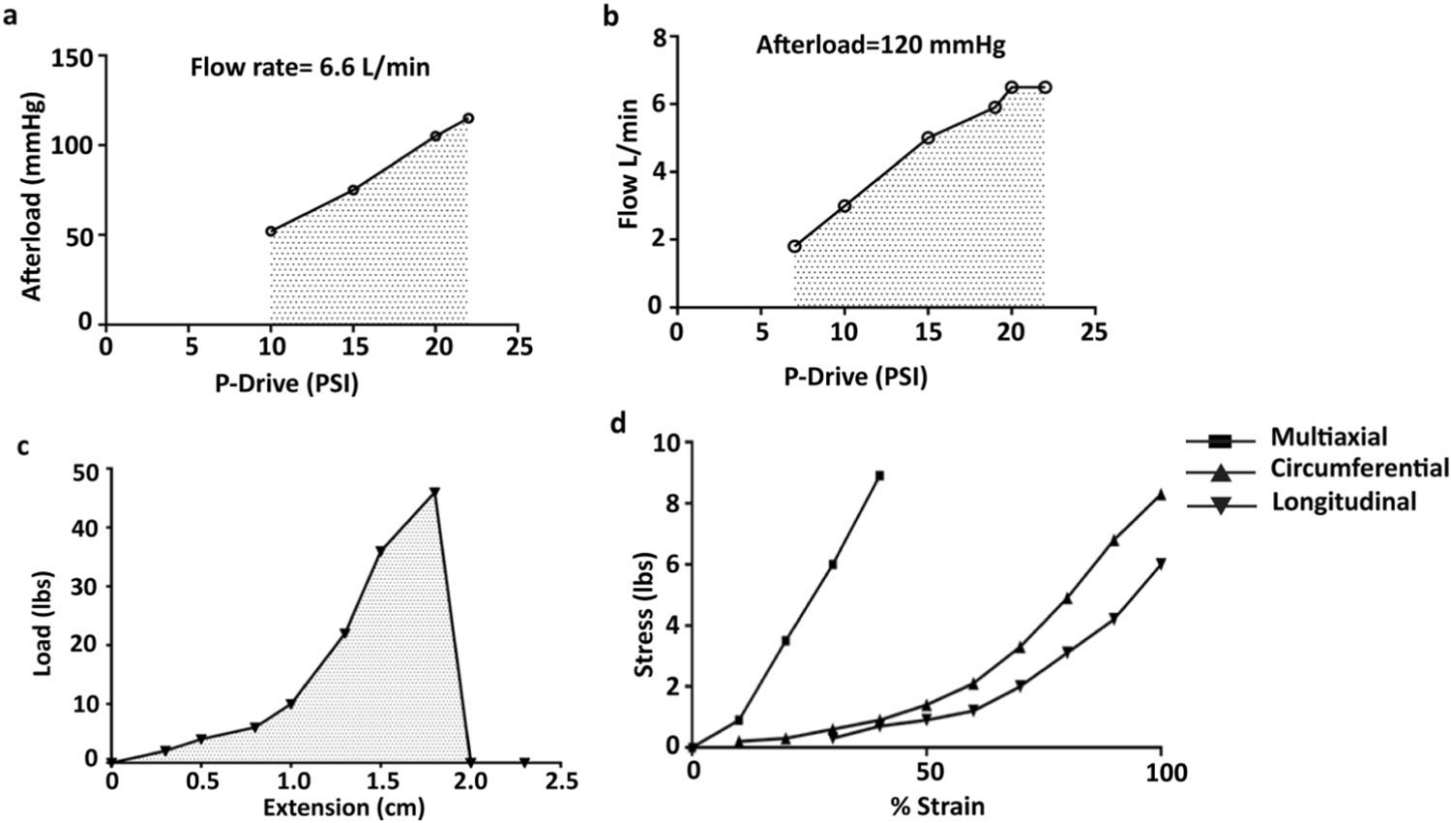
Functional characteristics of ASD device. **a** Pressure and **b** flow generated by ASD as a function of drive pressure for an afterload of 120 mmHg and an influx of 6.6 L/min, respectively. Calculations are shown as a solid curve. **c** Ball burst test of ASD, the pressure is given by pressing the ball at the center of ASD fixed in a circular fixture. ASD undergoes multiaxial expansion by the ball pressure. **d** Uniaxial circumferential and longitudinal curves were drawn, and a multiaxial curve was also drawn for comparison

The results of the pressure and flow characteristics of the ASD afterload were varied, and the driving pressure was adjusted to achieve a flow of 6.6 L/min (Fig. 2a). Similarly, constant afterload pressure was maintained while the driving pressure was changed to determine the device-generated flow (Fig. 2b). These in vitro measurements represent ASD operation for a complete failed ventricle.

#### Compliance characteristics of ASD device

The compliance experiments showed that ASD undergoes multiaxial expansion under load. A higher load resistance follows the early shallow part of the curve with a low resistance to load during the transition until ASD failure. The previous portion of the curve reaches 5 pounds (lb) and a 0.80 cm level; then, it becomes deformed and has a shallow slope. As the load increased above 5 lb, the slope becomes steeper. ASD reaches its load capacity and fails to function above the 46 lb load (Fig. 2c).

Figure 2d plot presenting the uniaxial compliance curve for ASD model oriented in the longitudinal and circumferential directions and multiaxial compliance curve for comparison. The slope of the multiaxial compliance curve is 4 times larger than the uniaxial curves between 25% and 45% strain. However, the extrapolated compliance curve is only 1.4–1.5 times greater than uniaxial compliance curves between 77% and 100% strain.

### In vivo experiments

#### Electrocardiography

**Fig. 3:**
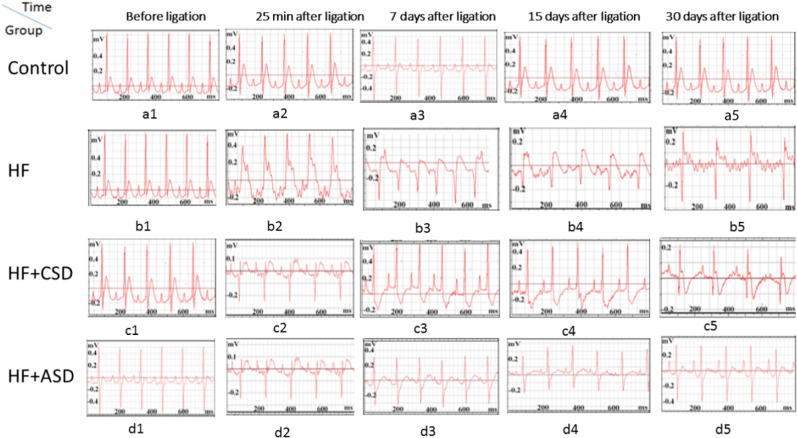
Electrocardiographic exploration of all rats’ groups during various periods. ECG contains a P-wave, followed by the QRS-complex and the T-wave. All measurement was recorded in a millisecond

All groups showed normal T-wave, no pathological Q-wave, isoelectric ST-segment, and upright T-wave before any surgery Fig. 3(a1, b1, c1, d1). ST-segment gets elevated early after 25 minutes of ligation in HF, HF + CSD, and HF + ASD groups Fig. 3(b2, c2, d2). HF group showed broad T-wave and smaller R-waves on the 7th and 15th-day Fig. 3(b3, b4), followed by pathological Q-waves and elevated ST-segment on 30th-day Fig. 3(b5). In the HF + CSD group, the T-wave starts smaller, and R-wave broadens on the 7th and 15th-day Fig. 3(c3, c4), however pathological Q-wave and elevated ST-segment emerged on 30th-day Fig. 3(c5). However, in the HF + ASD group T-wave get asymmetrical (upright), and R-wave deflects upward on the 7th and 15th-day Fig. 3(d3, d4), while on 30th-day smooth ST-segment (concave upward) and no pathological Q-wave was observed Fig. 3(d5).

#### Histopathology

**Fig. 4:**
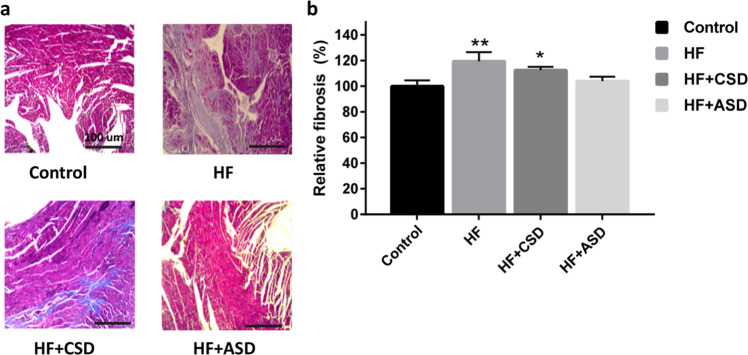
**a** Masson’s trichrome-stained section of rats’ heart in various groups by a photonic microscope (Original magnifications ×4). Showing a segment of the heart fibers encapsulated in collagen (blue) and overlying the myocardium (red). **b** Quantification of fibrosis of heart tissues obtained from different treatment groups, *n* = 3, Where, *P < 0.05, **P < 0.01, ***P < 0.001, scale bar 100 µm

Masson’s trichrome staining showed that the infarcted region of the collagen fibers and myocardium fibrosis appeared as blue color, while ordinary muscle fiber stained red. The control group was displayed free of a blue fibrotic area, while the HF group showed a distinct blue fibrotic area. While the HF + CSD showed less blue fibrotic area at the border zone, interestingly, the HF + ASD displayed a fibrotic-free blue area (Fig. 4a). In addition, the quantification of fibrosis by ImageJ software also showed similar observations (Fig. 4b).

#### Hemodynamic parameters

**Fig. 5:**
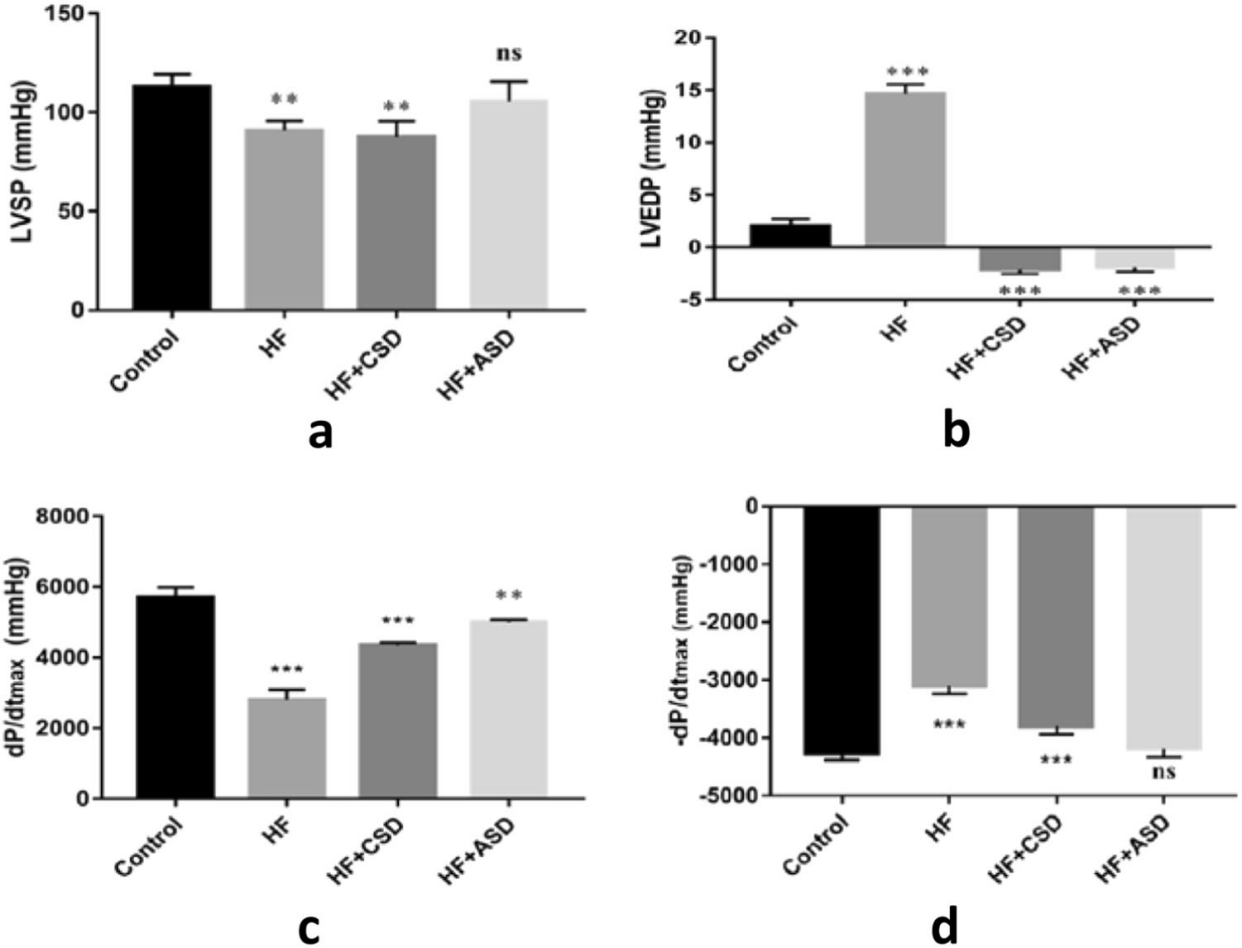
Hemodynamic data are presented as mean ± SEM (*n* = 6), 1way ANOVA followed by dunnetts’ multiple comparison tests was used for statistical significance of other groups compared to control group. **a** LVSP **b** LVEDP **c** dp/dt_max_ **d** −dp/dt_max_, ^ns^*P* = no significance;**P < 0.01; ***P < 0.001 vs. Control

**Fig. 6:**
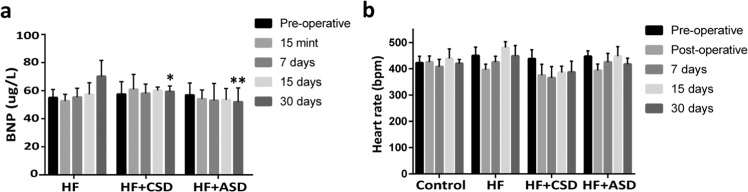
**a** The BNP results are expressed as mean ± SD; Two-way ANOVA followed by Bonferroni test was used to find statistical analysis compared to the HF group.*P < 0.01; **P < 0.001 vs HF group. **b** The heart rate of the control and treatment groups, HF, HF + CSD, HF + ASD groups. *n* = 3

Figure 5 illustrates the nature of restraint level on LVSP, LVEDP, and ventricle contractility assessment ±dP/dt_max_. As expected, LVSP decreased significantly in the HF group (89.74 mmHg) but has no significant difference in ASD treated (106.97 mmHg) compared with the control group. LVEDP level dropped in the HF + ASD group (−1.92 mmHg) and showed a significant difference as compared to the control group (1.88 mmHg), while in the HF group, it increased significantly (14.71 mmHg). dP/dt_max_ decreased in the HF group (*P* < 0.001) and increased in the ASD-treated group compared to the control group. Whereas −dP/dt_max_ showed a high significance value in HF and HF + CSD group (*P* < 0.001), and no significance in HF + ASD-treated rats was observed as compared with the control group. The heart rate of the HF + ASD group was brought to normal compared to supplementary treatment groups (Fig. 6a).

#### Plasma BNP levels

Coronary artery ligation resulted from the increased plasma BNP levels in all groups. BNP level was evaluated at different stages during the treatment period. After the placement of the restraint device, subsequently, plasma BNP levels dropped in ASD (51.983 μg/L) and CSD (59.462 µg/L) treated rats but didn’t show any significant difference till the end of the treatment period as compared to HF (70.125 μg/L) group. However, at the end of the one-month treatment period, plasma BNP level decreased in HF + ASD significantly (*P* < 0.001) as compared to the HF group (Fig. 6a).

## Discussion

Ventricular restraint therapy is a promising therapeutic approach for the treatment of end-stage HF. Previous studies on animals and human beings showed CSD (Acorn CorCap) and Paracord HeartNet devices could reverse the pathologic LV remodeling [10, 11]. However, for recommencing interest and further optimization of restraint therapy, it requires a comprehensive understanding of the precise relationship of restraint nature by optimizing its design and biocompatibility of VRT with the host heart. Previous studies of CorCap CSD applied latex-free polyester mesh for delivering standard restraint and reversing pathologic LV remodeling [11, 14].

In vitro studies using pneumatic drive showed the functional ability of ASD devices. The unique characteristic of the ASD device is volume amplification, in which the hydraulic stroke volume needed to actuate the device is smaller than the resulting blood stroke volume. The required volume amplification is achieved for higher drive pressure as a result of the conservation of energy. The physiological afterload pressure and the stroke volume are equal to or less than the hydraulic drive pressure and the hydraulic stroke volume [33]. The functional ability of the ASD is in the normal range, which is vital for normal heart function (Fig. 2a, b).

How the ASD is fitted to the heart and the heart shape can also influence compliance and device-related fibrosis [34]. A comparative degree of movement intertwined within the ASD device can be observed during mechanical loading. When the ASD device was examined against multiaxial stress-strain study and material was exposed to a load up to about 5 pounds per inch. Generally, the expansion of the material remained between 12% and 22%. The compliance value under initial loading permits the ASD device to conform closely to the heart surface at the time of implantation and prevents unnecessary load development at end-diastole [35]. The outward force exerted by the heart during diastolic filling is relatively less than 5 pounds of equivalent burst load [36]. Therefore, in actual use conditions, the multiaxial extension of the ASD remains within the shallow part of the multiaxial stress-strain curve. Compliance in the longitudinal direction is somewhat more significant than the circumferential direction (Fig. 2c, d), which promotes a chronic shape modification of the heart from spherical to a more ellipsoid shape [34, 37]. Multiaxial compliance was shown to be lower than individual uniaxial compliance values. This indicates that the limiting stiffness of the ASD in multiaxial or uniaxial loading is similar. However, strain to reach that restraint is reliant on the loading direction.

Indeed, the electrocardiography showed numerous (Fig. 3) abnormalities in HF. The most frequently seen electrocardiographic abnormality is broad T-waves, small R-waves, deep Q-waves, and ST-elevation, characteristic features of dilated ventricular and MI [38]. These abnormalities were observed in the HF group on the 7^th^, 15^th^, and 30th-day Fig. 3(b3–b5), similarly it was noted in CSD-treated rats on 30th-day Fig. 3(c5). ASD showed improvement in treating dilated ventricle, ventricular relaxation, and relieving HF symptoms characterized by smooth ST-segment, upright T-wave, and normal Q-wave Fig. 3(d4, d5) [39, 40]. ASD device was found safe and feasible regarding the electrical activity of the heart; furthermore, it improved cardiac performance and did not alter the structure and function of the myocardium.

Masson’s trichrome staining is a valuable histopathologic tool and commonly used to differentiate collagen fibers from tissues and muscles. The acidophilic cytoplasm of myocardial tissues was stained with an acidic dye. Collagen, a comparatively loose texture, is easily passable by most dyes. However, after the following treatment, the dye will quickly diffuse out, permitting collagen to be stained with aniline blue and give a blue-stained collagen fiber [41]. Cardiac cell death occurs after LAD coronary artery ligation [42] and becomes noticeable in the HF group on the 30th day. Left ventricle remodeling after LAD ligation is categorized by hypertrophy and fibrotic changes to the heart [43]. The death of cardiac cells leads to a decrease in contractile force [44]. The CSD-treated rats showed a blue stain at the border zone, which may be due to the reaction of an attached device with the heart. Whereas SD rats treated with ASD device showed no blue-stained area (Fig. 4).

In the current study on the 30th day, the hemodynamic parameters were assessed, which revealed that LVSP and −dp/dt_max_ were decreased while LVEDP and +dp/dt_max_ were increased significantly in the HF group as compared to the control group. This indicates that both systolic and diastolic function in HF rats were impaired [45]. However, after ventricle restraint therapy, these pathological changes were restored. The result showed that hydraulic restraint (HF + ASD) leads to a greater and earlier reduction in LVEDP than standard restraint (HF + CSD). LVEDP reflects the LV’s compliance and ability to receive blood from the left atrium during diastole [46].

Moreover, when the LV compliance decreases, the LVEDP rises, which initiates MI, ventricle dilatation, and another cascade of HF [47, 48]. Furthermore, LVSP and -dp/dt_max_ positively correlate with systolic function, and it showed no significant difference compared to the control group after hydraulic restraint (Fig. 5) [48, 49]. Besides, ASD is feasible, biocompatible, and didn’t impair the hemodynamic parameters. It delivers hydraulic pressure to the ventricle, not only at the systolic but also at the diastolic phase throughout the cardiac cycle.

To further confirm the morphological and functional changes, the molecular marker of HF at various periods during the study was evaluated (Fig. 6a). The level of BNP increased with the development of HF after coronary artery ligation. It is produced in response to myocardial stretch and ventricle dilatation due to pressure or volume overload [50]. Till mid of the treatment period, rats treated with ASD showed a slight decline in BNP level but not significantly different. However, at the end of the treatment period, the ASD + HF group is substantially diverse from the HF group.

Nonetheless, the current study demonstrated that hydraulic restraint provided by an ASD device could lead to the decreased BNP-associated remolding. To summarize, the HF group showed both systolic and diastolic dysfunction. Hydraulic restraint (HF + ASD) leads to a more significant reduction in LV trans-myocardial pressure and ventricular wall stress, leading to more considerable reverse remodeling than standard restraint. It might be one possible mechanism by which restraint applied through ASD prompts reverse remolding at a molecular level.

## Conclusions

To sum up, the ASD device is structurally composed of silicon, a non-immunogenic and biocompatible material, and provides promising restraint therapy compared to previous standard restraint therapies. It improves cardiac function and reverses ventricular remodeling. Therefore, the future aim of current research is to deliver biological therapeutic agents to the heart through ASD, and this initial feasibility study will prove an impetus to move forward. However, further studies are needed to verify whether pathologic remodeling persists or not after the termination of restraint therapy? What are the effect of ASD implantation on the ventricular shape, size, and myocardial structure, as well as load-independent indices of ventricular functions over a long treatment period?
